# Hearing of neonates without risk indicators for hearing loss and use of antimalarial drugs during pregnancy: a historical cohort study in the Northern Region of Brazil

**DOI:** 10.1016/j.bjorl.2019.06.005

**Published:** 2019-07-23

**Authors:** Fernanda Soares Aurélio Patatt, André Luiz Lopes Sampaio, Pedro Luiz Tauil, Carlos Augusto Costa Pires de Oliveira

**Affiliations:** a Universidade Federal de Santa Catarina (UFSC), Curso de Fonoaudiologia, Florianópolis, SC, Brazil; b Universidade de Brasília (UnB), Ciências da Saúde, Brasília, DF, Brazil; c Universidade de Brasília (UnB), Faculdade de Medicina, Brasília, DF, Brazil; d Universidade de Brasília (UnB), Faculdade de Medicina, Programa de Pós-Graduação em Medicina Tropical, Brasília, DF, Brazil; e Universidade de Brasília (UnB), Brasília, DF, Brazil

**Keywords:** Malaria, Antimalarials, Hearing, Newborns, Pregnancy

## Abstract

**Introduction:**

Studies have demonstrated the ototoxic effects of antimalarial drugs in individuals who receive these drugs, but little is known regarding the toxicity of these drugs in the newborn auditory system when administered to the mother receive the drug during pregnancy.

**Objective:**

To verify the incidence of hearing loss in neonates who have no other associated risk indicators, born to mothers treated for malaria during pregnancy.

**Methods:**

A retrospective, quantitative cohort study was developed at Hospital de Base Dr. Ary Pinheiro and Clínica Limiar, both located in the municipality of Porto Velho (Rondônia). The sample consisted of 527 newborns divided into two groups: exposed to antimalarials drugs during pregnancy group (n = 32) and non-exposed group (n = 495). Data collection took place from September 2014 to December 2015, through an interview with the mothers and/or guardians of the newborn, through the newborns’ and the mothers’ records, and the neonatal hearing screening database of the above-mentioned institutions.

**Results:**

All the neonates in the exposed group, assessed through the recording of transient otoacoustic emissions associated with the automated brainstem auditory evoked potential test, underwent neonatal hearing screening in the first examination. Among the newborns in the non-exposed group, 30 showed failure and were retested. Of these, one continued to fail and was referred for diagnosis, in whom the results showed to be within the normal range. Among the neonates of the exposed group, infection with *Plasmodium vivax* was the most frequent, and was similarly distributed among the gestational trimesters, and chloroquine was the most commonly used antimalarial drug treatment more often given during the third trimester; these findings did not show any influence on the audiological findings of the studied neonates.

**Conclusion:**

The present study did not identify any cases of hearing loss in neonates born to mothers who used antimalarial drugs during gestation.

## Introduction

Malaria is the most important endemic infectious disease in the Brazilian Amazon region, due to its wide dissemination in the area, high incidence, impact on morbidity and mortality and difficultly to control.^1^

Of the nine states that constitute the Legal Amazon region (states of Acre, Amapá, Amazonas, Maranhão, Mato Grosso, Pará, Rondônia, Roraima and Tocantins), Rondônia ranks second and third in recorded cases of malaria between 2002 and 2011.^2^

In 2006, the municipalities of Cruzeiro do Sul (AC), Manaus (AM) and Porto Velho (RO) accounted for 22.59% of the total cases of malaria in the Amazon region.^2^

The clinical picture of malaria can be classified as mild, moderate or severe according to the parasite species, the number of circulating parasites, the duration of disease and the level of immunity acquired by the patient.^3^ In severe and complicated malaria, some clinical and laboratory characteristics should be highlighted. These include: altered consciousness, bleeding, jaundice, severe anemia, hypoglycemia, renal failure,^3^, ^4^ dyspnea or hyperventilation, seizures, hypotension or shock, pulmonary edema, and others.^3^ Along with infants and those infected for the first time, pregnant women are most severely affected, especially if infected by *Plasmodium falciparum*; early and correct diagnosis and timely treatment are the most appropriate means to reduce malaria severity and lethality.^3^

Among the complications of maternal malaria during conception and delivery, studies have recorded cases of miscarriage,^5^, ^6^ stillbirths,^6^ prematurity^5^, ^7^, ^8^, ^9^ and low birth weight.^5^, ^7^, ^8^, ^9^, ^10^, ^11^

The number of positive slides in pregnant women in the municipality of Porto Velho in 2010 totaled 331 cases combining rural and urban areas, including 48 cases of P. falciparum, 280 cases of *Plasmodium vivax* and 3 cases of mixed malaria (*P. falciparum* + *P. vivax*).^12^

Studies have demonstrated the ototoxic effects of antimalarials in individuals who use these drugs;^13^, ^14^, ^15^, ^16^, ^17^, ^18^ however, little is known about their toxicity to the auditory system of neonates when ingested by mothers during pregnancy.

Studies have reported that when chloroquine crosses the placenta, it may be ototoxic to fetuses.^14^, ^19^ A study carried out in 2014 at the referral public hospital in the capital of Rondônia, describing the results of the neonatal hearing screening (NHS) program concerning maternal infection with a risk of vertical transmission, showed that malaria was the most frequent infection (35.4%) in the mothers whose children were treated by the program. The NHS failure was 1.5%.^20^

A study carried out in 2004, confirmed 12 cases of hearing loss in children whose mothers had been treated with antimalarials during pregnancy. All the children were diagnosed with sensorineural hearing loss, seven of them profound and five between moderate and severe.^21^

In Porto Velho (RO), there were two cases (11.8%) of hearing loss in children from zero to three months of age whose mothers used antimalarials during pregnancy. Both children were diagnosed with unilateral mild sensorineural hearing loss.^22^

A study carried out in the NHS program of the referral public hospital of the state of Rondônia showed that the neonates of mothers treated for malaria during pregnancy had a percentage of failure in the NHS program of 6.7% that was 5.64 times higher than the newborns (NBs) of untreated mothers^23^; another study, carried out in the same program, showed hearing loss in 3% of the NBs of mothers treated for gestational malaria.^24^ However, the few existing publications^22^, ^23^, ^24^, ^25^ do not isolate the antimalarial use factor in the gestational period from the other risk indicators for hearing loss (RIHL) in children.

The need to evaluate the effect of antimalarial use in the gestational period on the hearing of the newborns without the possibility of the influence of other RIHL justifies the present study. Based on the above, the aim was to verify the frequency of hearing loss in neonates of mothers treated for malaria during pregnancy, without other associated RIHL, as well as to characterize the exposed group (EG) and the non-exposed group (NEG) to antimalarials in the gestational period regarding the variables: age, origin and maternal schooling, as well as gestational age (GA) and birth weight; to characterize the EG regarding the type and trimester of the malaria infection and the type of antimalarial drug used and the gestational trimester when the medication was administered.

## Methods

This is an epidemiological, observational, analytical, historical cohort study, evaluated and approved by the Research Ethics Committee (REC) with human subjects under number 314,929. The present research was carried out at Hospital de Base Dr. Ary Pinheiro (HBAP) and at Clínica de Avaliação e Reabilitação da Audição — Limiar, both located in the city of Porto Velho (RO).

All newborns in this study whose mothers did not receive antimalarial treatment during the gestational period were screened with Transient Evoked Otoacoustic Emissions (TEOAE), and neonates who failed the test were retested within 15 days. The newborns whose mothers received antimalarial drugs, as well as those who did not receive them, but failed the evaluation with TEOAE, were screened using the combined technique (TEOAE + Automated Brainstem Auditory Evoked Potential − A-BAEP). Data collection for this study occurred between September 2014 and December 2015.

The following eligibility criteria were established for case selection: newborns of Brazilian mothers, born at HBAP in rooming-in care, without RIHL, or only with the antimalarial factor during pregnancy, submitted to all phases of the NHS program at the time of data collection and have performed all recommended steps for neonatal hearing screening program,^26^ whose mothers received an adequate prenatal care and agreed to participate in the study, signing the Free and Informed Consent Form (FICF).

The sample was estimated based on the mean number of newborns in rooming-in care screened at the HBAP during the data collection period, which is 3600. Adopting a 5% error and a level of significance of 95%, a minimum sample size of 348 newborns was estimated.

Prior to hospital discharge, 734 mothers or guardians of newborns in rooming-in care admitted to the HBAP at the time of data collection were approached, who agreed to participate in the study by authorizing access to the medical records of the mother and the newborn. Of these, 173 were excluded because they had RIHL, 25 because they did not perform all the recommended steps for the NHS programs (7 newborns had failed the NHS, were referred for diagnosis and failed to come) and 9 because the mothers did not undergo prenatal care.

Based on the above, the sample comprised 527 newborns, who were divided into two groups, namely: exposed group (EG) and non-exposed group (NEG). The first group consisted of 32 newborns of mothers who used antimalarial drugs during the gestational period and the second of 495 newborns with no risk of hearing loss in childhood.

Aiming at isolating the variable “use of antimalarials in the gestational period” and to avoid the presence of confounding variables, only newborns without RIHL were included in NEG, whereas the EG included newborns born to mothers who used antimalarials during the gestational period with no other associated RIHL.

Of the 32 neonates that comprise the EG, 17 were females (53.1%) and 15 males (46.9%) and of the 495 neonates in the NEG, 251 were females (50.7%) and 244 (49.3%) were males.

The newborns from the EG had a mean GA of 38.4 weeks and a mean birth weight of 3131 g, while the newborns from the NEG had a mean GA of 38.6 weeks and a mean birth weight of 3229 g.

Data collection started with an interview with the newborns’ parents or guardians to obtain socioeconomic and demographic data, family history of hearing loss in childhood, and confirm the information that was subsequently obtained from the medical records, such as the occurrence of congenital infections and prenatal care and, in the cases of mothers who had gestational malaria, information on the type of infection, period of occurrence, drug treatment, and the period during which the medication was administered (first, second and/or third trimester of pregnancy) was obtained.

In addition to the interview, the newborn’s and the mother’s medical files, the HBAP NHS database, the Clínica Limiar database, the GN Otometrics MADSEN AccuScreen® portable device, the newborn’s vaccination card, the pregnant woman's card and data collection form were used for data collection.

All compiled data were recorded in the data collection form, in order to guarantee the organization and quality of the data collection, tabulated in Excel spreadsheets and submitted to statistical tests with a significance level of 5% (*p* ≤ 0.05).

The ANOVA statistical test was used to characterize the sample regarding the quantitative variables (maternal age, birth weight and GA), as well as to compare these variables between the groups. To analyze the distribution of the qualitative variables (maternal level of schooling and origin and newborn gender) as well as to compare such variables between the groups, the statistical test of equality of two proportions was used. This statistical test was also used to characterize the subjects of the EG, regarding the type and gestational trimester of malaria infection, as well as to verify the different antimalarial drugs used during the gestational period, as well as the gestational period of their administration, in addition to comparing the TEOAE results, according to the ear, between the groups and compare the results of the NHS tests between the groups.

In addition to these analyses, it was possible to calculate the relative risk of the newborns from both groups to fail the TEOAE in the left ear, whereas it was not possible to perform the same analysis regarding the TEOAE in the right ear and the final result of the NHS, since no failures were obtained in the right ear at the TEOAE and in the final result of the NHS in the EG.

We documented infection in five mothers who were infected in more than one gestational period; the gestational period of malaria infection in the mothers treated with Coartem (artemether + lumefantrine) and Quinine + Clindamycin; the number of mothers treated for malaria after the infectious period and the treatment received according to the gestational trimester (one or more trimesters).

Finally, the results of the audiological evaluation of the newborns referred for diagnosis were described.

## Results

The age of the mothers who had malaria during the gestational period ranged from 14 to 39 years, with a mean of 23.3 years and mothers who were not affected by the disease ranged from 14 to 43 years, with a mean of 25.1 years. These values, when compared, did not show any statistically significant difference (*p* = 0.140).

It was demonstrated, in both groups, that the significant majority of the mothers came from Porto Velho (RO) (*p* < 0.001). Among the mothers of the newborns from the EG, 24 come from the capital of Rondônia, six from the countryside of the state and two from the countryside of the neighboring state (Amazonas). Among the mothers of the newborns from the NEG, 336 were from Porto Velho, 154 from the countryside of the state and 5 from the countryside of the neighboring state (Amazonas).

Regarding the level of schooling, most mothers from the EG (56.3%; n = 18) reported not having finished Elementary School, a significant finding when compared to the other levels of schooling: Complete Elementary School — CES, Incomplete High School — IHS, Incomplete College/University — IC/U and Complete College/University — CC/U (*p* < 0.0001); Complete High School — CHS (*p* = 0.044).

Among the NEG mothers, the majority reported having completed high school (34.6%, n = 171), a finding that did not differ statistically only from the number of mothers who did not complete elementary school (31.2%, n = 154, *p* = 0.250), indicating a significant difference when compared to other levels of education (CES, IHS, IC/U and CC/U, *p* < 0.001).

When comparing the variables GA and newborn birth weight between the groups, no significant difference was observed (Table 1).

**Table 1:** Comparison of the variables gestational age and birth weight between the groups.

	Mean	Median	SD	CV	Min.	Max.	N	CI	*p*-Value
GA (weeks)									
Exposed	38.4	38.5	1.6	4%	34.5	41.1	32	0.6	0.418
Non-exposed	38.6	39	1.6	4%	33	42.3	493	0.1	
Birth weight (grams)									
Exposed	3.131	3.200	431	14%	2.406	3.900	32	149	0.315
Non-exposed	3.229	3.270	540	17%	1.780	4.860	495	48	

Statistical test: ANOVA (*p* ≤ 0.05).

GA, gestational age; SD, standard deviation; CV, coefficient of variation; Min., minimum; Max., maximum; CI, confidence interval; N, number; %, percentage; *p*-value, level of statistical significance.

*P. vivax* infection was the most frequent in the study population, showing a statistically significant difference when compared to the occurrence of *P. falciparum* infection (Table 2).

**Table 2:** Distribution of the newborns regarding the type and period of infection by malaria.

Type of infection	N	%	*p*-Value	Period of infection	N	%	*p*-Value
*P. vivax*	25	78.1	<0,001^a^	1^st^ Trimester	14	43.8	Ref.
*P. falciparum*	7	21.9		2^nd^ Trimester	13	40.6	0.800
				3^rd^ Trimester	12	37.5	0.611

Statistical test: Equality of two proportions (*p* ≤ 0.05).

EG, exposed group; N, sample number; %, percentage; 1st, first; 2nd, second; 3rd, third; Ref., reference; p-value, level of statistical significance.

a Statistical significance.

The distribution of infection among the gestational trimesters was quite homogeneous, with the first trimester being the most recurrent, but without statistical difference when compared to the others (Table 2). It is noteworthy that five mothers were infected in more than one gestational period, with one of them being infected in the first and second gestational trimesters, two in the first and third trimesters and two throughout the gestational period (all three trimesters), with all five being infected by *P. vivax.*

The antimalarial drug most frequently used by the mothers of the newborns in the EG was chloroquine, a statistically significant finding when compared to the use of Coartem (artemether + lumefantrine), with the treatment performed with quinine associated with clindamycin, and primaquine (Table 3).

**Table 3:** Distribution of the newborns of the EG regarding the type of antimalarial drug used and the period of administration.

Antimalarial drug	N	%	*p*-Value	Period of infection	N	%	*p*-Value
Chloroquine	27	84.4	Ref.	1st Trimester	14	43.8	0.005^a^
Coartem (artemether + lumefantrine)	3	9.4	<0.001^a^	2nd Trimester	19	59.4	0.106
Quinine + Clindamycin	2	6.3	<0.001^a^	3rd Trimester	25	78.1	Ref.
Primaquine	1	3.1	<0.001^a^				

Statistical test: equality of two proportions (*p* ≤ 0.05).

EG, exposed group; N, sample number; %, percentage; 1st, first; 2nd, second; 3rd, third; Ref., reference; *p*-value, level of statistical significance.

a Statistical significance.

The three mothers treated with Coartem (artemeter + lumefantrine) were infected by *P. falciparum*, with two of them infected in the second trimester and one in the third trimester of pregnancy. Of the two women treated with quinine associated with clindamycin, both were infected in the first trimester, but one was infected by *P. vivax* and the other by *P. falciparum*.

Moreover, three mothers infected with *P. falciparum* reported having been treated with chloroquine, one of whom used this antimalarial drug in combination with primaquine.

The drug treatment with antimalarials was more frequent in the third trimester, and this result was significant when compared to antimalarial administration in the first trimester of pregnancy (Table 3). Fourteen mothers (43.75%) reported having received the treatment beyond the period(s) of disease occurrence, according to medical indication.

Three mothers (9.38%) underwent treatment only in the first trimester, whereas another 3 mothers (9.38%) underwent treatment only in the second trimester, and 8 mothers (25%) underwent treatment only in the third trimester. One mother (3.12%) reported having used antimalarial drugs in the first and second gestational trimesters, 2 (6.25%) reported treatment in the first and third trimesters, 7 (21.87%) reported treatment in the second and third trimesters and 8 (25%) were treated for malaria during the three gestational trimesters.

When the isolated TEOAE results were analyzed according to the tested ear, it was verified, in the first test, that no EG newborn failed during the right ear evaluation, whereas one newborn failed the left ear test. On the other hand, 21 of the newborns of the NEG failed the TEOAE in the right ear and 29 in the left ear. These findings showed statistical significance when the intragroup comparison was performed, but no statistically significant difference was observed when comparing the findings between the groups (Table 4). No association was observed between TEOAE failure in the left ear and antimalarial exposure during pregnancy (Table 5).

**Table 4:** Comparison between TEOAE results according to the tested ear in the first test.

		TEOAE RE				TEOAE LE			
		Passed	Failed	Total	*p*-Value	Passed	Failed	Total	*p*-Value
Exposed	N	32	0	32	<0.001^a^	31	1	32	<0,001^a^
	%	100%	0%	100%		96.9%	3.1%	100%	
Non-exposed	N	474	21	495	<0.001^a^	466	29	495	<0,001^a^
	%	95.8%	4.2%	100%		94.1%	5.9%	100%	
*p*-Value		0.234				0.518			

Statistical test: Equality of two proportions (*p* ≤ 0.05).

TEOAE, transient evoked otoacoustic emissions; RE, right ear; LE, left ear; N, sample number; %, percentage; *p*-value, level of statistical significance.

a Statistical significance.

**Table 5:** Relative risk of left ear TEOAE failure in neonates exposed to antimalarial drugs during the gestational period.

TEOAE LE	Failed	Passed	Total	RR
Exposed	1	31	32	0.53 (0.08^a^ 3.58)
Non-exposed	29	466	495	
Total	30	497	527	

TEOAE, transient evoked otoacoustic emissions; LE, left ear; RR, relative risk.

a Statistical significance.

There was a higher incidence of TEOAE failure in the left ears of newborns from the NEG, indicating that the use of antimalarials in the gestational period may protect the hearing of newborns (Table 5). However, this result was not statistically significant.

The newborns from the EG who showed TEOAE failure in the left ear were not retested, since they showed a satisfactory response in the A-BAEP test, thus “passing” the NHS.

Thus, only the newborns who showed “failure” in the first test and belonged to the NEG, which were evaluated only through TEOAE, underwent the retest. Of the 30 newborns who underwent TEOAE retest, only one patient continued to show “failure” (bilateral) and was then referred for diagnostic evaluation.

All newborns from the EG, evaluated through the combined technique (TEOAE + A-BAEP), passed the NHS immediately after the first examination. Of the 495 newborns that comprised the NEG, evaluated only through TEOAE, as they were considered of low risk for hearing loss, 33 showed “failure” at the first test, and of these, three underwent A-BAEP and passed, with retesting not being necessary.

Of the 30 retested newborns, 53.3% (n = 16) were retested before hospital discharge and 46.6% (n = 14) were reassessed 15 days after the first test, i.e., after hospital discharge. After retesting, only one newborn persisted showing “failure” at the test, which was bilateral.

Thus, of the 32 newborns from the EG, all passed the NHS (100%) and of the 495 from the NEG, 494 passed the NHS (99.8%), with the newborn that failed being referred to diagnostic evaluation.

This neonate came for diagnostic evaluation at 22 days of age and showed absence of diagnostic TEOAE in both ears; presence of waves I, III and V in the diagnostic BAEP, with absolute and interpeak latencies within the normal range, demonstrating integrity of the auditory pathway to the high brainstem; electrophysiological threshold at the frequency of 2000 Hz within normality (30 dB) and normal mobility of the ossicular-tympanic system (type A tympanometric curve), bilaterally. Considering these results, and the fact that this newborn did not have RIHL, he was discharged from the NHS program and the mother was instructed regarding the auditory and linguistic development of the child.

Therefore, none of the 527 newborns who participated in the present study (EG = 32; NEG = 495) were diagnosed with hearing loss.

## Discussion

The present study did not demonstrate any cases of hearing loss in neonates born to mothers who used antimalarial drugs (Chloroquine, Coartem, Quinine and Primaquine) during pregnancy. This finding corroborates that found by Silva (2014), which characterized the NHS program of the hospital where this study was developed, which verified that although the occurrence of malaria during the gestational period is the infection with the most frequent risk of vertical transmission in the studied population, none of the infants diagnosed with hearing impairment had a history of gestational malaria.^27^

Malaria during pregnancy was also a risk indicator with a significant occurrence in neonates under rooming-in care, in a study carried out with newborns from private health units in the city of Porto Velho (RO); however, the neonates exposed to gestational malaria did not have hearing loss.^28^

Another study, carried out in the same municipality, diagnosed 15 newborns with hearing impairment (2/1000 newborns), but no cases of antimalarial use were detected in the gestational period among the diagnosed neonates.^29^

On the other hand, other studies verified hearing loss in children exposed to antimalarials in the gestational period^21^, ^24^, ^25^; however, none of them isolated the use of antimalarials in the gestational period from other RIHL, since the diagnosed children had other risk indicators associated with malaria treatment during pregnancy, which could have been responsible for the identified alterations.

Therefore, there is still no evidence that “antimalarial use during pregnancy” constitutes a risk factor for hearing loss in neonates.

Moreover, the present study did not show a significant difference in GA and in the weight of neonates from both groups (exposed and non-exposed to antimalarials during the gestational period), with these findings being quite similar to those found by other researchers.^24^

It is believed that the mothers of the present study who had malaria during pregnancy received an early diagnosis and timely treatment, with no alterations in the course of gestation, as well as in GA and birth weight.

This result also confirms the findings of a study carried out in Thailand with 300 pregnant women, totaling 376 episodes of malaria, of which 246 were of *P. falciparum* infection, treated with quinine and 130 episodes of *P. vivax* infection, treated with chloroquine, which found that there was no increase in rates of congenital abnormalities, fetal death or low birth weight. According to the researchers, these results suggest that the therapeutic doses of chloroquine and quinine are safe.^30^ On the other hand, other studies have reported cases of premature birth^5^, ^7^, ^9^ and low birth weight.^5^, ^7^, ^9^, ^10^, ^11^

Another hypothesis that may justify the absence of alterations during the course of gestation and, consequently, in the GA and in the weight of the newborns of the present study is the fact that the identified malarial episodes were not classified as severe or complicated ones.

In contrast, a study carried out in Ibadan, Southwest Nigeria, found an association between maternal malaria during pregnancy and the newborn’s birth weight (*p* = 0.007); showing significantly lower birth weight in the newborns from the group exposed to malaria during gestation.^31^

Regarding the maternal data, it was observed that the mean age of the mothers of the newborns in the EG corroborates that found in a study carried out with neonates of women who used antimalarials in the gestational period, showing a mean maternal age of 23.1 years^24^ and in a study developed with 417 pregnant women who had malaria during pregnancy and underwent drug treatment.^32^ The mean age of women who did not have malaria during pregnancy and, therefore, did not undergo drug treatment, was older (25 years) and was compatible with that observed in studies carried out in the NHS programs in Recife (PE).^33^, ^34^

Most of the mothers who participated in this study come from the capital city of Rondônia, as well as verified in recent studies developed in the same hospital.^24^, ^27^

As for the level of schooling, most of the mothers in the EG did not finish elementary school, while most mothers from the NEG had finished high school, a finding that agrees with that reported in other studies carried out in the Amazon region,^24^, ^27^, ^32^ and shows that the higher the level of schooling, the lower the malaria infection rate. In contrast, the study by Dræbel et al. suggested that the level of schooling does not have to be high for people to undergo malaria prevention and treatment; however it found an association between school attendance with malaria diagnosis and treatment.^35^

The present study showed that infection by *P. vivax* was the most prevalent, as was shown in other studies^5^, ^9^, ^10^, ^20^^,^^23^, ^24^, ^25^, ^32^, ^36^ carried out in the same municipality or in neighboring regions, most of which are part of the legal Amazon region. However, studies carried out in India,^6^ Nigeria^37^, ^38^ and Thailand^30^ showed that infection by *P. falciparum* was more frequent.

Regarding the gestational period when the infection occurred, no difference was observed in the distribution between the three gestational trimesters, a finding that corroborates that of other studies developed in the same program,^23^, ^25^ but it is opposite to that observed in a study carried out in the same hospital, which found a higher frequency of infection in the first trimester^24^ and studies carried out in Hospital da Mulher Mãe Luzia, in the municipality of Macapá (AP),^36^ and in a prenatal clinic in Benin City, Nigeria, which showed a higher occurrence of malaria in the third trimester of pregnancy.^8^

In agreement with the recommendations by the Ministry of Health, the most commonly used antimalarial in the treatment of *P. vivax* infection in the present study was chloroquine.^3^, ^4^, ^39^ Other studies have also emphasized the use of chloroquine for malaria caused by *P. vivax*,^20^, ^22^, ^23^, ^24^, ^25^, ^30^, ^32^, ^36^ and in a study of 199 pregnant women carried out in Nigeria, chloroquine was the most frequently used drug for the treatment of *P. falciparum* malaria.^8^

Of the seven cases of *P. falciparum* infection reported in this study, three mothers had malaria caused by *P. falciparum* in the second and third trimesters of pregnancy and were treated with Coartem (artemether + lumefantrine), which is recommended for pregnant women with *P. falciparum* malaria in the second and third trimesters.^4^, ^39^ Other authors also found Coartem as the preferred treatment for *P. falciparum* malaria in the second and third gestational trimesters.^36^

Only two mothers were treated with quinine together with clindamycin, one infected by *P. vivax* and the other with *P. falciparum*, both in the first trimester of pregnancy, the treatment recommended by the Ministry of Health for cases of severe and complicated malaria caused both by *P. falciparum* and *P. vivax* in the first gestational trimester.^4^ This finding coincides with a study carried out in Thailand, with women treated for malaria in the first trimester of pregnancy.^30^

It was also reported that three mothers were treated for *P. falciparum* infection with chloroquine, which is contraindicated because this parasite is resistant to the aforementioned drug.^39^ The association of chloroquine and primaquine was used to treat a mother infected with *P. falciparum* in the first trimester of pregnancy, although primaquine is contraindicated because it can result in fetal hemolysis.^3^ A study published in 2010 indicates primaquine as one of the most commonly used antimalarial drugs in the treatment of gestational malaria. Additionally, it reports that primaquine has a gametocidal action against *P. falciparum,* but it is contraindicated during pregnancy due to undesirable effects on the fetus, such as methemoglobinemia.^40^

Although most of the treatments performed are in agreement with recommendations of the Ministry of Health,^3^, ^4^, ^39^ there was no consensus regarding the therapy used according to the gestational trimester, as also verified in a study carried out in Macapá (AP).^36^ Moreover, there was no agreement regarding the treatment of malaria according to the type of infection, since three mothers infected with *P. falciparum* were treated with chloroquine, a drug that is not effective against this *Plasmodium* due to its resistance to the medication.

In the present study, drug treatment with antimalarial drugs was more frequent in the third trimester, a result that corroborates findings from other studies^8^, ^22^; however, it disagrees with those from a study that found that treatment for malaria was more frequent in the second trimester, although no significant difference was found when compared to treatment in the other gestational periods.^24^ It is believed that in the present study, malaria treatment was more frequent in the third trimester, since in addition to the women infected in that trimester (n = 12, 37.5%), many of those infected in the first and second trimesters reported having been treated until the neonate’s sixth month of life.

As for the auditory evaluation of the neonates, the first test showed a higher number of failures in the left ear, as previously shown in other studies with newborns,^41^, ^42^ a result that may be explained by the fact that the OAE amplitude is lower in this ear.^43^, ^44^, ^45^

All newborns exposed to antimalarial drugs in the gestational period were screened using TEOAE associated with A-BAEP, as recommended for neonates at high risk for hearing loss.^46^ Due to the knowledge of the antimalarials’ ototoxic propensity and lack of evidence of the ototoxic effect of the antimalarials used by the mother during pregnancy on the neonate, the program carried out at HBAP classifies the newborns exposed to antimalarials in the gestational period as a risk for the development of hearing loss. Despite this, all 32 newborns from EG showed normal A-BAEP results in 35 dBHL, and therefore they passed the NHS, demonstrating the absence of auditory impairment in neonates exposed to antimalarials (Chloroquine, Quinine, Coartem – artemether + lumefantrine – and Primaquine) in the gestational period, at the moment of the auditory evaluation of this study participants.

In contrast to the results obtained in the present study, Silva et al. demonstrated a 6.7% failure rate in the NHS of newborns whose mothers had been treated for malaria during pregnancy, showing they were 5.64 times more likely to fail in NHS than the NB of mothers not treated for gestational malaria.^23^ Similarly, Aurélio et al. showed 11.4% (n = 4) of cases of NHS failures in neonates whose mothers used antimalarials during the gestational period.^24^ It is noteworthy that, in both of those studies, the NHS of the newborns exposed to antimalarials during pregnancy was performed using the combined technique (OAE and A-BAEP), as was the case in the present study. It is believed that this divergence is due to the fact that the mentioned studies^23^, ^24^ did not isolate the use of the antimalarials in the gestational period from the other RIHL, which may have contributed or potentiated the results found.

We suggest the need for new studies, with a larger number of subjects, a situation that was not feasible in this study because of the changes that occurred in the HBAP NHS program at the time of data collection, which currently screens all newborns only through OAE.

Moreover, performing a case-control study might also be appropriate because hearing loss, as well as malaria infection, are not frequent. However, in this situation, case-control studies could have a bias due to the fact that the neonates in the group (with hearing loss) might show other RIHL associated with treatment for malaria during pregnancy and hinder establishing the association between hearing loss and the use of antimalarials during pregnancy, as the alterations observed in this group may be due to the other RIHL observed in the neonates, thought to have occurred in other previous studies.^22^, ^24^, ^25^

It is believed that the findings of the present study, related to GA, newborn birth weight, as well as the absence of auditory alterations in neonates exposed to antimalarial drugs in the gestational period, are explained by the adequate and timely treatment, as proposed by the Ministry of Health.^4^

We also suggest that the hearing of these newborns be monitored and studies with children and adolescents exposed to antimalarial drugs in the gestational period, be developed aiming to investigate the occurrence of progressive hearing loss in this population. A study of 725 children and adolescents from Mozambique who received antimalarials, identified 12 subjects, with a mean age of 3.2 years who received antimalarials in the gestational period, and were identified with sensorineural hearing loss, seven of them with profound and five with moderate to severe hearing loss.^21^ It is noteworthy that the mothers of these children diagnosed with hearing loss were treated for the most part with Chloroquine and Chloroquine associated with Quinine,^21^ antimalarials also used by the mothers of the present study.

## Conclusion

The present study of neonates with no other associated RIHL did not identify any cases of hearing loss in neonates born to mothers who used antimalarial drugs (Chloroquine, Coartem, Quinine and Primaquine) during pregnancy, with all neonates from the EG successfully going through NHS tests with TEOAE and A-BAEP since the first test.

Based on these facts, it is necessary to develop studies that aim to monitor the hearing of children exposed to antimalarial drugs in the gestational period, preferably without other RIHL, aiming to investigate the occurrence of progressive hearing loss in this population.

## Conflicts of interest

The authors declare no conflicts of interest.
